# CBCT assessment of maxillary molar distalization and root length changes during clear aligner therapy

**DOI:** 10.1186/s12903-026-08896-1

**Published:** 2026-06-26

**Authors:** Ahmed Ibrahim Atalla, Noha Hussein Abbas, Noha Ezzat Sabet, Marwah Salah Abd El-Latif

**Affiliations:** 1 Orthodontic Department, Ain-Shams University, Cairo, Egypt; 2 Orthodontic Department, Ahram Canadian University, Cairo, Egypt

**Keywords:** Clear aligner, Molar distalization, CBCT, Class II malocclusion, OIIRR

## Abstract

**Background:**

This prospective CBCT case series evaluated the pattern of maxillary molar movement during sequential distalization with clear aligners and vertical attachments, without adjunctive Class II elastics or skeletal anchorage, and assessed associated orthodontically induced inflammatory root resorption.

**Methodology:**

Ten maxillary quadrants from seven adult patients with Class II molar relationships were included. All patients were treated using a standardized sequential molar distalization protocol with clear aligners. CBCT scans were obtained at baseline (T0) and immediately after completion of molar distalization (T1). Linear crown and root positional changes, as well as molar root length changes, were assessed using three-dimensional CBCT superimposition. Descriptive statistics and paired t-tests were used, with significance set at *P* < 0.05.

**Results:**

The maxillary first and second molars demonstrated significant distal displacement at the mesiobuccal cusp tips: 1.24 ± 0.64 mm and 1.02 ± 0.82 mm, respectively. Root apex displacement was limited, suggesting crown-dominant distal movement rather than true bodily distalization. The maxillary central incisors showed significant labial incisal-edge displacement of 1.93 ± 1.27 mm, while root apex position remained stable, indicating partial anterior anchorage loss under the tested protocol. Small but statistically significant reductions in molar root length were detected, ranging from 0.19 ± 0.21 mm to 0.47 ± 0.68 mm.

**Conclusions:**

Within the limitations of this exploratory prospective case series, sequential maxillary molar distalization with clear aligners and vertical attachments produced modest crown-dominant distal molar movement. Mild anterior anchorage loss was observed under a protocol without adjunctive elastics or skeletal anchorage. Molar root length reductions were small and remained below 1 mm. Larger controlled studies incorporating planned vs. achieved movement analysis are required.

## Introduction

Maxillary molar distalization is one of the most common approaches for treating Class II molar malocclusion, referring to the distal movement of the molars to create space within the dental arch. Ideally, distal molar movement should occur as bodily movement, in which the crown and root move simultaneously. This contrasts with tipping movement, where only the crowns shift while the root apex remains relatively stationary [1]. To achieve true bodily movement, the applied force must be directed through the molar’s center of resistance, which has been reported to lie slightly occlusal to the furcation of the maxillary molar roots [2]. 

A wide variety of appliances have been developed to facilitate distalization of the maxillary molars. Extraoral headgear has long been considered effective; however, its effectiveness is highly dependent on patient compliance. To overcome this limitation, several intraoral appliances—such as the Pendulum, Distal Jet, and Jones Jig—have been introduced. Although these appliances reduce reliance on patient cooperation, they generally cannot produce pure bodily movement, as the applied force vector seldom aligns precisely with the molar’s center of resistance, often resulting in significant distal tipping [3]. In fixed appliances, achieving bodily movement requires a carefully controlled force system that includes both an active force and an opposing counter-moment to minimize unwanted tipping [1]. 

Clear aligner therapy (CAT) has recently emerged as an increasingly popular orthodontic modality. Early theoretical models suggested that aligners would primarily induce tipping or intrusion, with limited potential for generating bodily tooth movement [4]. However, experimental studies have demonstrated that aligners can achieve bodily movement when appropriate biomechanical conditions are employed [5]. Notably, maxillary molar distalization has been identified as one of the most predictable tooth movements with clear aligners, with an accuracy of approximately 87%, surpassing movements such as torque and derotation [6, 7]. The use of attachments has been shown to enhance the efficiency of distalization further [8, 9]. 

Despite these promising findings, available evidence assessing the three-dimensional effects of sequential maxillary molar distalization in adult patients treated exclusively with clear aligner therapy, without adjunctive elastic use, remains limited. A more comprehensive understanding of these biomechanical effects is needed, particularly with respect to the pattern of tooth movement and the potential development of orthodontically induced inflammatory root resorption (OIIRR).

Therefore, this prospective CBCT case series aimed to evaluate the pattern of maxillary molar movement and associated root length changes following sequential distalization with clear aligners and vertical attachments in adult patients treated without adjunctive Class II elastics or skeletal anchorage.

## Materials and methods

### Study design, ethics, and recruitment

This study was approved by the institutional ethics committee, Research Ethics Committee, Faculty of Dentistry, Ain Shams University, Cairo, Egypt (Approval No. FDASU-Rec ID 032254). The study was also registered in the National Library of Medicine database (Protocol No. ClinicalTrials.gov (http://clinicaltrials.gov/) Identifier: NCT06330571).; Registration Date: 17 March 2022). Written informed consent was obtained from all participants before enrollment. All procedures involving human participants were conducted in accordance with the ethical standards of the institutional research committee and with the 1964 Declaration of Helsinki and its later amendments or comparable ethical standards. Participants who met the inclusion criteria and were consecutively recruited between 2023 and 2025 underwent maxillary molar distalization using clear aligner therapy (CAT). Patients were included without pre-selection to minimize selection bias. Only cases with cone-beam computed tomography (CBCT) records at baseline (T0) and immediately after completion of distalization (T1) were included in the final analysis. CBCT scans were obtained as part of the routine diagnostic and treatment evaluation protocol, and no additional radiographic exposure was performed solely for research purposes.

### Study sample and imaging protocol

The study sample consisted of 10 maxillary quadrants from seven adult patients (six females and one male; age range: 18–24 years) presenting with an Angle Class II molar relationship requiring maxillary molar distalization. Only quadrants requiring active distalization were included, and in unilateral Class II cases, only the treated quadrant was analyzed.

CBCT scans were acquired using a Planmeca Promax 3D Mid scanner (Planmeca Oy, Helsinki, Finland) with a field of view of 80 × 50 mm, voxel size of 0.2 mm, and exposure parameters of 90 kV, 8 mA, and 12 s. Baseline (T0) and post-distalization (T1) scans were used for three-dimensional assessment of tooth movement and associated apical root resorption.

### Eligibility criteria

The inclusion criteria were: (1) permanent dentition, (2) age ≥ 18 years, (3) Class II molar relationship requiring maxillary molar distalization, and (4) good compliance with aligner wear. Maxillary third molars were extracted before the distalization protocol to eliminate potential posterior interference with molar movement. Exclusion criteria included periodontal disease, severe skeletal discrepancies, absence of permanent maxillary teeth (excluding third molars), and prosthodontic rehabilitation of the maxillary molars.

### Sample size calculation

A priori sample size calculation was performed based on the expected difference between the predicted and actual maxillary molar distalization, which was used as a measure of treatment accuracy. Based on a previous study [10], the mean predicted distalization was 2.42 ± 1.2 mm, and the actual distalization was 0.88 ± 0.83 mm. To ensure a conservative estimate, the larger standard deviation (1.2 mm) was used. Accordingly, a minimum sample size of 10 molars was required to detect a clinically relevant difference of 1.2 mm with 80% power at a significance level of α = 0.05 using a paired t-test. The sample size calculation was performed using PS Power and Sample Size Calculation software (PS Power version 3.1.2 for MS Windows, developed by William D. Dupont and Walton D. Plummer, Vanderbilt University, USA).

### Treatment protocol

All patients were treated with clear aligners by the same operator using an identical sequential molar distalization protocol without the use of elastics or other auxiliary anchorage devices. No overcorrection was planned. Patients were instructed to wear the aligners full-time, except during meals, and to change them every 14 days, with a mean treatment duration of 41 weeks, in accordance with standard clinical practice. This allowed for sufficient biological tooth movement while minimizing adverse effects. The 14-day aligner change interval was intentionally selected to ensure complete expression of programmed distalization movements, optimize aligner tracking, and allow adequate stress relaxation of the aligner material [11–13]. The right and left maxillary quadrants of each patient were analyzed separately to capture potential side-specific responses, while acknowledging that teeth within the same quadrant may not be fully independent. At each visit, the operator evaluated the fitness of the aligners and the integrity of the attachments. Proper aligner seating was used as an indirect indicator of patient compliance with the prescribed wear protocol [14]. 

### Digital planning and biomechanics

**Fig. 1:**
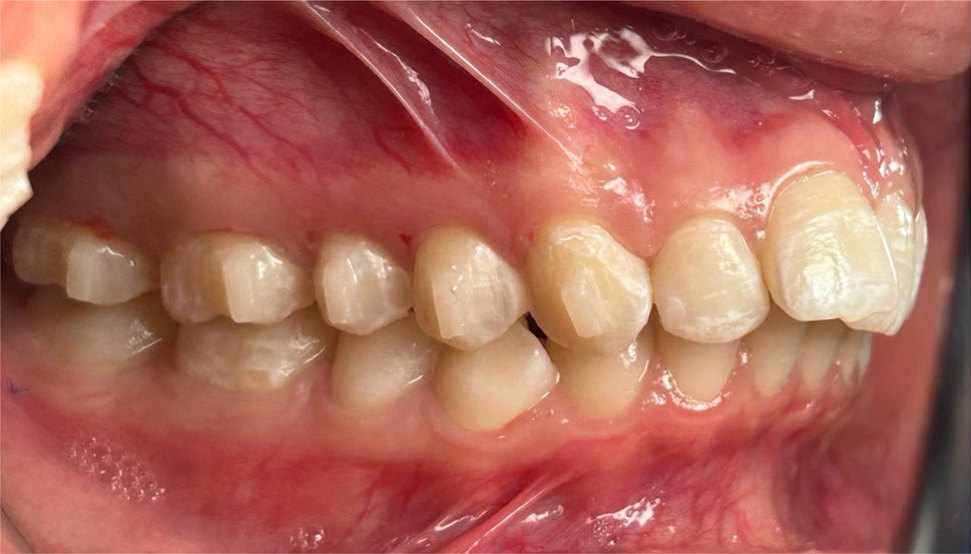
Placement of vertical attachments on the maxillary teeth

ThreeShape software was used to generate a virtual setup and aligner staging. The amount of distalization was determined individually for each case based on the initial molar relationship to achieve a Class I molar relationship; however, quantitative planned movement values per tooth were not extracted for analysis. Sequential distalization was designed in three stages with a staging of 0.25 mm, beginning with the second molar. Once the second molar had completed two-thirds of its planned movement, the first molar was activated for distalization [7–9]. No specific molar derotation was intentionally programmed as part of the standardized distalization protocol. Vertical attachments were placed from the maxillary canine to the second molar to enhance control during distalization and reinforce anchorage of the premolars and anterior teeth, thereby reducing the risk of unwanted mesial movement and incisor flaring, following the protocol described by Garino et al. [8] Attachments were centered mesiodistally and occlusogingivally, measuring 2.75 × 1.75 × 1.0 mm [15]. In cases presenting with unilateral Class II molar relationships, only the quadrant requiring distalization was included in the analysis. CBCT imaging was performed before treatment (T0) and immediately after molar distalization (T1) to assess dental changes (Fig. 1).

### Aligners fabrication

Aligners were fabricated using single-layer thermoformed 0.75-mm Duran sheets [16, 17]. A MINISTAR S pressure-forming machine was used to thermoform the sheets according to the manufacturer’s protocols, utilizing barcode-guided settings with a working pressure of 4 bar to ensure accurate adaptation and a consistent thickness of 0.75 mm.

### Measurement protocol

Linear positional changes of the maxillary molars, premolars, and central incisors were assessed radiographically using CBCT [14]. 

**Fig. 2:**
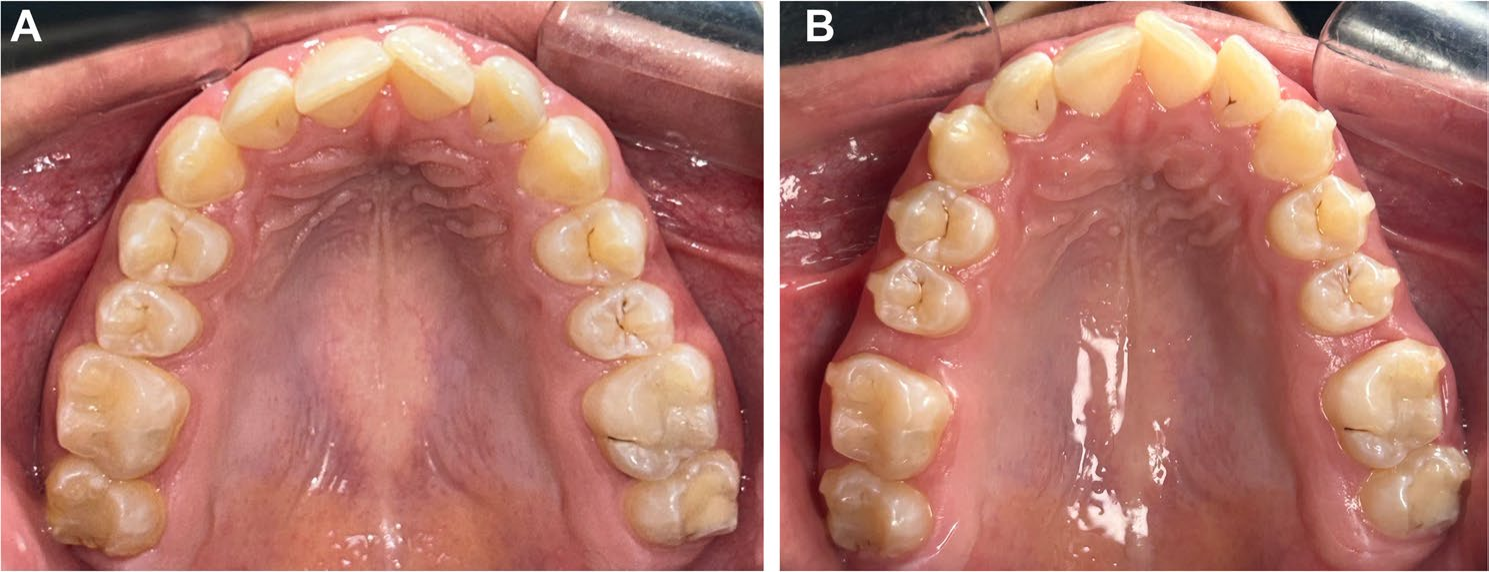
CBCT scans at baseline (T0, **A**) and immediately after completion of molar distalization (T1, **B**)

**Fig. 3:**
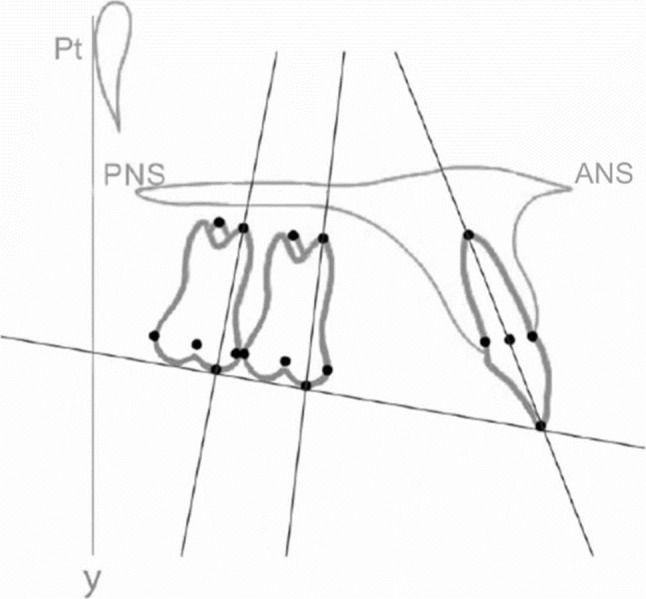
Schematic illustration of linear tooth movements relative to the pterygoid vertical (PTV) plane

CBCT scans obtained at baseline (T0) and immediately after distalization (T1) were analyzed digitally. The average duration between T0 and T1 scans was approximately six months (Fig. 2). Linear tooth movements were measured using Invivo 5 software (Anatomage, San Jose, CA, USA) following best-fit three-dimensional superimposition of pre- and post-treatment scans with automatic volume-based registration, which has been validated for accurate and reproducible assessment of tooth displacement [18, 19]. The pterygoid vertical (PTV) plane served as a reference for scan alignment, while actual measurements were performed in the mesiodistal dimension of both the crown and root of each tooth (Fig. 3). Although measurements were limited to the mesiodistal axis, the use of CBCT and best-fit superimposition ensured precise spatial alignment and reliable assessment of distal tooth movements. This approach allows accurate quantification of tooth displacement and associated root changes while minimizing operator-dependent errors, providing objective data on the biomechanical effects of maxillary molar distalization with clear aligners.

**Fig. 4:**
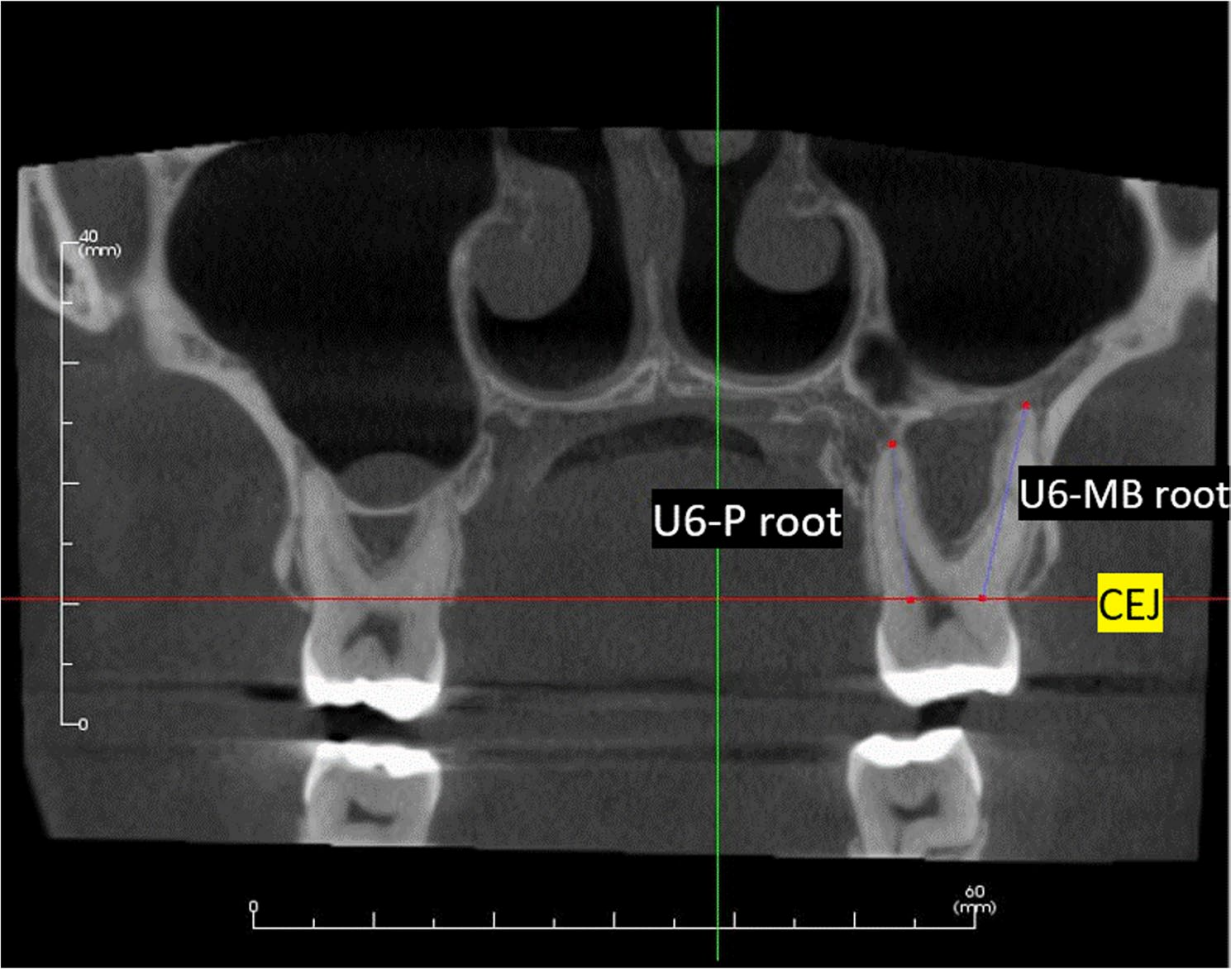
Measurement of root length changes from the cemento-enamel junction (CEJ) to the root apex

Root length changes of the maxillary molars, representing OIIRR, were assessed using the same software. Coronal sections were oriented as illustrated in Fig. 4. For the mesiobuccal root, the vertical dental axis (VDA) was defined from the mesiobuccal cusp tip to the root apex, while for the palatal root, the VDA extended from the palatal cusp tip to the palatal root apex. The cemento-enamel junction (CEJ) was identified in the same view, and root length was measured as the linear distance from the CEJ to the corresponding root apex. Linear root length measurements obtained from CBCT images have been widely used to evaluate orthodontically induced root resorption and provide a reliable method for detecting changes in root length between time points [20]. 

### Statistical analysis

All measurements were performed by a single examiner. To assess intra-examiner reliability, 10 quadrants were randomly selected and remeasured after a 14-day interval. Reliability was evaluated using the intraclass correlation coefficient.

Descriptive statistics were calculated for all variables. Pre- and post-distalization measurements were compared using paired t-tests, with statistical significance set at *P* < 0.05. Because quadrants rather than patients were used as the unit of analysis, the findings should be interpreted as exploratory, and possible intra-patient correlation should be considered when interpreting statistical significance.

## Results

A total of 10 maxillary quadrants from seven patients were analyzed. Overall, patients demonstrated adequate cooperation with the aligner wear. Moderate compliance was observed in one patient, whereas the remaining six patients exhibited good compliance.

**Fig. 5:**
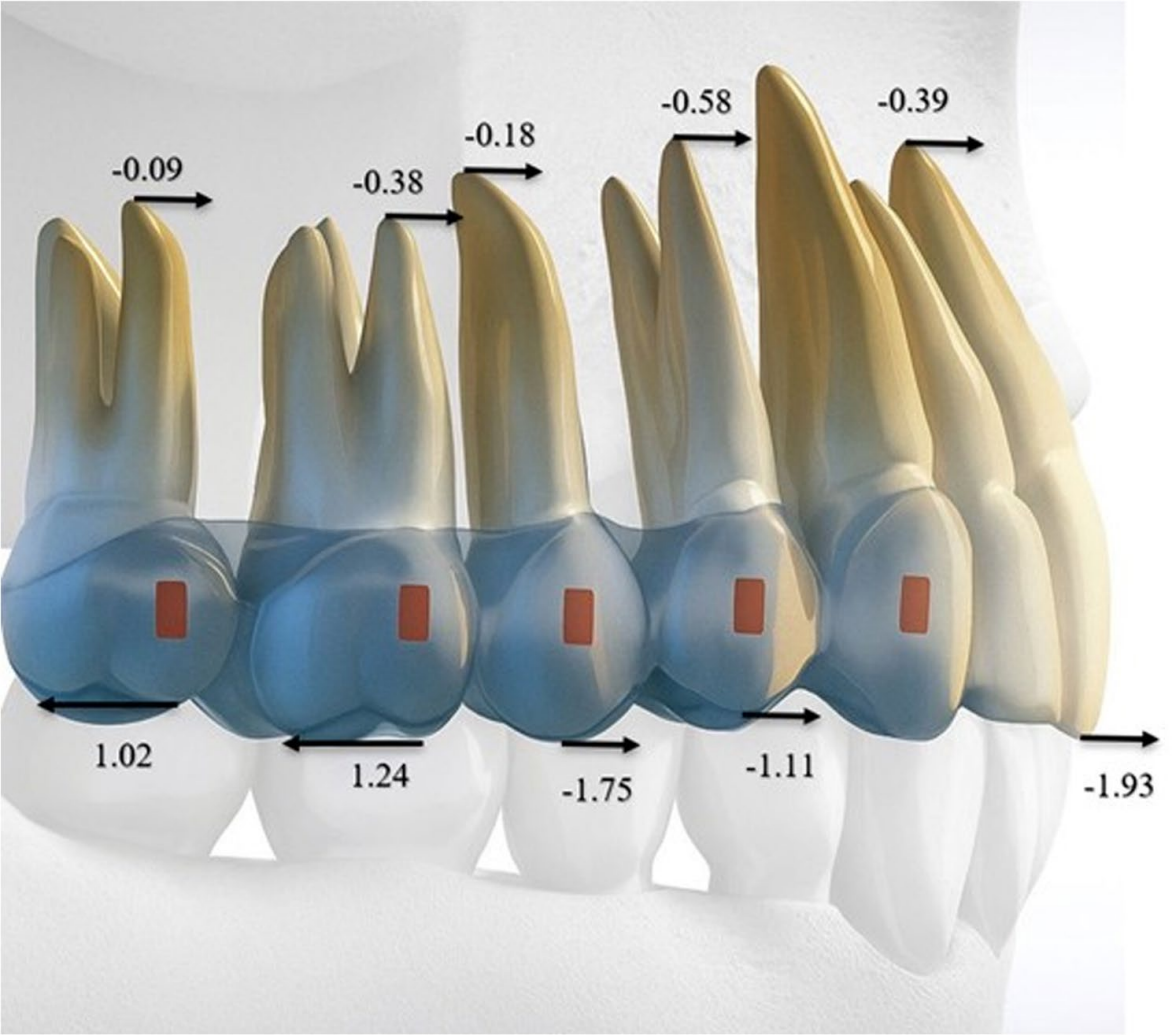
Schematic representation of clinically relevant linear changes observed in this study

Descriptive statistics for the dental variables referenced to the pterygoid vertical plane (PTV), measured by the pretreatment and post-distalization CBCT scans, are presented in Table 1. Significant sagittal positional changes of the maxillary molars were observed following distalization. The maxillary second molar demonstrated a statistically significant distal movement of 1.02 ± 0.82 mm at the mesiobuccal cusp, while the mesiobuccal root apex remained stable. Similarly, the maxillary first molar showed a statistically significant distal movement of 1.24 ± 0.64 mm at the mesiobuccal cusp, with no significant change in the position of its mesiobuccal root apex. The maxillary second premolars demonstrated a statistically significant mesial movement of 1.75 ± 0.87 mm, indicating partial loss of posterior anchorage during distalization. In contrast, the maxillary first premolars exhibited non-significant positional changes. The maxillary central incisor edge showed a statistically significant protrusion of 1.93 ± 1.27 mm, whereas its root apex remained unchanged in position. (Fig. 5).

**Table 1:** Descriptive statistics and statistical comparison of the T1–T0 changes with a paired t-test

Variable	T0 (Pre-treatment) (mm)		T1 (post-treatment) (mm)		T1–T0 Change (mm)				*P*-Value	
	**Mean**	**SD**	**Mean**	**SD**	**Min**	**Max**	**Mean**	**SD**		
U7-PTVMC	13.53	1.86	12.51	2.41	-0.69	2.12	1.02	0.82	**0.013**	*
U7-PTV-MB root	15.46	2.13	15.6	2.14	-2.51	3.77	-0.092	1.64	**0.635**	NS
U6-PTVMC	24.81	1.67	23.56	2.1	-0.23	2.12	1.249	0.64	**0.007**	*
U6-PTV-MB root	24.36	2.33	24.74	2.72	-2.28	1.06	-0.38	0.9	**0.208**	NS
U5-PTV-B	29.51	2.61	31.26	2.94	-3.43	-0.45	-1.752	0.87	**0.005**	*
U5-PTV-B root	30.16	3.05	30.34	3.76	-2.29	1.65	-0.183	1.16	**0.553**	NS
U4-PTV-B	36.6	2.05	37.71	3.05	-2.98	1.64	-1.11	1.44	**0.059**	NS
U4-PTV-B root	35.01	2.48	35.59	3.03	-2.98	0.46	-0.581	1.07	**0.128**	NS
U1-PTV-II	53.28	4.53	55.22	4.98	-3.43	0.91	-1.938	1.27	**0.007**	*
U1-PTV-Root	45.71	2.3	46.1	2.4	-1.82	0.23	-0.393	0.79	**0.261**	NS

Values are shown as mean & SD (standard deviation). Positive values indicate distal tipping. Negative values indicate mesial tipping

*P < 0.05 (statistically significant), NS (Not significant)

Regarding root length changes, the maxillary first molar exhibited a statistically significant reduction of 0.30 ± 0.32 mm in the mesiobuccal root and 0.19 ± 0.21 mm in the palatal root. The maxillary second molar also showed significant root length reduction of 0.47 ± 0.68 mm in the mesiobuccal root and 0.27 ± 0.27 mm in the palatal root (Table 2).

**Table 2:** Root resorption (RR) using CBCT

ROOT RESORPTION ON CBCT	Before treatment		After treatment		Change				*P*- Value	
	**Mean**	**SD**	**Mean**	**SD**	**Min**	**Max**	**Mean**	**SD**		
RR-U6-MB root	11.71	2.26	11.41	2.1	0	1.01	0.3	0.32	**0.008**	*
RR-U6-p root	11.77	1.37	11.58	1.24	-0.25	0.5	0.19	0.21	**0.037**	*
RR-U7-MB root	11.65	1.94	11.18	2.3	-0.36	2.13	0.47	0.68	**0.028**	*
RR-U7-p root	11.36	1.29	11.09	1.51	-0.2	0.65	0.27	0.27	**0.021**	*

*NS* Not significant

*P < 0.05 (statistically significant)

## Discussion

Maxillary molar distalization is considered one of the more predictable tooth movements achievable with clear aligner therapy. Simon et al. [7] reported that the accuracy of upper molar distalization using aligners was approximately 87%, with at least 1.5 mm of predicted distal movement when virtual models were analyzed. In contrast, studies by Ravera et al., [9] Garino et al., [8] and Caruso et al. [14] evaluated molar distalization using lateral cephalograms and concluded that the upper molars could be bodily distalized by 2–3 mm.

In the present study, the first molar achieved 1.24 mm of distalization, slightly more than the 1.02 mm observed for the second molar, with the mesiobuccal root apices of both teeth remaining stable. This pattern may be explained by the biomechanics of sequential distalization with clear aligners, where force is transmitted continuously across the arch rather than as isolated activations. When the first molar begins its programmed distalization after the second molar, interaction forces within the aligner system can result in slight mesial movement or reduced resistance of the second molar while the first molar continues to move distally. Such interaction effects have been described in biomechanical and finite element studies of aligner mechanics, in which distalizing forces applied to one tooth generate reciprocal forces on adjacent units, reflecting the continuous nature of the aligner force system [21, 22]. Although these distalization amounts were modest, the primary objective of this exploratory case series was to evaluate the pattern of tooth movement and associated root resorption rather than to achieve complete Class II correction. Direct comparison with previous studies is limited due to methodological differences. For example, Simon et al. [5] used a retrospective design with varying treatment stages, and distalization was calculated after a series of aligners. Ravera et al. [8] incorporated Class II elastics in combination with aligners, which may have enhanced molar movement. Garino et al. [8] conducted a case–control study using lateral cephalometric radiographs, while Caruso et al. [14] also relied on cephalometric evaluation in a retrospective study. In contrast, the present study utilized CBCT in a prospective design, offering a more accurate evaluation of the pattern of molar movement and associated root changes. However, differences in study design and the absence of adjunctive mechanics in the present protocol should be considered when interpreting these comparisons.

Several previous studies using CBCT have evaluated molar distalization with clear aligners, although most were retrospective and varied in methodology [18, 20, 23]. For example, Elfouly et al. [23] reported changes in molar inclination and rotation using CBCT, highlighting the complexity of achieving controlled tooth movement with aligners. Similarly, Al-Worafi et al. [20] investigated alveolar bone changes and orthodontically induced inflammatory root resorption, demonstrating the value of CBCT in assessing both mechanical and biological effects. More recent CBCT-based analyses using three-dimensional superimposition have also evaluated the accuracy of distalization, further emphasizing discrepancies between planned and achieved tooth movement [18]. Compared with these studies, the present prospective design provides additional insight into the pattern of molar movement and associated root changes under controlled clinical conditions. However, a direct comparison remains limited due to differences in study design and the use of adjunctive mechanics.

A significant finding in the current study was the 1.93 mm protrusion of the maxillary central incisors, while the root apex remained stable. This proclination likely resulted from reciprocal forces generated during molar distalization, particularly since no adjunctive anchorage mechanics (e.g., Class II elastics or skeletal anchorage) were used beyond vertical attachments. Consistent with previous reports [8, 9, 24], the use of Class II elastics during distalization has been suggested to help control anterior flaring, especially in patients presenting with increased overjet. Our findings align with Saif et al., [25] who reported anchorage loss of approximately 39.9% at the upper central incisors during molar distalization. However, as no comparison group with adjunctive mechanics was included in the present study, the potential role of elastics in improving anterior anchorage control cannot be directly inferred from the present data and remains speculative.

Regarding root resorption, previous investigations have suggested that clear aligner therapy may be associated with a lower risk of OIIRR, possibly due to the intermittent and relatively light forces exerted by removable appliances [26–28]. In the present study, statistically significant but small reductions in root length were observed: 0.30 mm (mesiobuccal root) and 0.19 mm (palatal root) for the first molar, and 0.47 mm (mesiobuccal root) and 0.27 mm (palatal root) for the second molar. These changes are relatively minor and are likely to be of limited clinical relevance. To our knowledge, only one study by Al-Worafi et al. [20] has evaluated molar OIIRR following clear aligner therapy, reporting average reductions of 0.45 mm in the first molar and 0.32 mm in the second molar, which are consistent with the findings of the present study. According to Elfouly et al., [19] OIIRR below 1 mm in maxillary molars is generally considered clinically insignificant. Variations in the magnitude of OIIRR reported across studies may be attributed to differences in imaging modalities, measurement protocols, sample size, aligner material properties, and the magnitude and duration of orthodontic forces applied.

The present study has several limitations. First, the relatively small sample size and prospective case-series design limit the generalizability of the findings. Although the sample size was statistically justified based on prior data, the results should be interpreted as exploratory in nature. Second, quadrants rather than patients were used as the unit of analysis; therefore, possible intra-patient correlation and biomechanical interaction between right and left sides cannot be excluded, particularly as clear aligners function as a continuous arch system with force distribution across the dentition. Third, only quadrants requiring molar distalization (including unilateral Class II cases) were included, and non-distalized quadrants were not used as internal controls, which may introduce selection bias. Fourth, although distalization was digitally planned to achieve a Class I molar relationship, quantitative planned tooth movement values were not consistently retrievable from the digital setup; therefore, planned-versus-achieved movement and accuracy ratios could not be calculated. Finally, angular measurements of molar tipping, torque, and rotation were not included, limiting definitive interpretation of the exact type of tooth movement. Future studies with larger sample sizes and controlled study designs are recommended to confirm these findings and to further investigate the adjunctive use of Class II elastics with aligners for minimizing unwanted anterior effects during maxillary molar distalization.

## Conclusions

Within the limitations of exploratory prospective CBCT case series:

1. Sequential maxillary molar distalization with clear aligners and vertical attachments produced modest distal displacement of the maxillary first and second molar crowns.
2. Limited root apex displacement suggests a crown-dominant distal movement pattern rather than confirmed bodily distalization.
3. Labial displacement of the maxillary central incisor edge was observed, suggesting partial anterior anchorage loss under the tested protocol without adjunctive elastics or skeletal anchorage.
4. Molar root length reductions were small and remained below commonly reported clinically relevant thresholds (< 1 mm).
5. Future controlled studies with larger samples, cluster-adjusted statistical models, planned vs. achieved movement analysis, and angular CBCT assessment are required to further validate and expand these findings.

## Data Availability

The data sets produced and reviewed in this study are not publicly accessible due to patient privacy concerns; however, they can be obtained from the corresponding author upon reasonable request.
